# Distinguishing Syntactic Operations in the Brain: Dependency and Phrase-Structure Parsing

**DOI:** 10.1162/nol_a_00029

**Published:** 2021-02-01

**Authors:** Alessandro Lopopolo, Antal van den Bosch, Karl-Magnus Petersson, Roel M. Willems

**Affiliations:** Department of Psychology, University of Potsdam, Germany; Center for Language Studies, Radboud University, Nijmegen, Netherlands; Meertens Institute, Royal Netherlands Academy of Science and Arts, Amsterdam, Netherlands; Max Planck Institute for Psycholinguistics, Nijmegen, Netherlands; Center for Language Studies, Radboud University, Nijmegen, Netherlands; Max Planck Institute for Psycholinguistics, Nijmegen, Netherlands; Donders Institute for Brain, Cognition, and Behaviour, Radboud University, Nijmegen, Netherlands

**Keywords:** neural basis of syntactic processing, dependency grammar, phrase structure grammar, functional MRI, anterior temporal pole, superior temporal gyrus

## Abstract

Finding the structure of a sentence—the way its words hold together to convey meaning—is a fundamental step in language comprehension. Several brain regions, including the left inferior frontal gyrus, the left posterior superior temporal gyrus, and the left anterior temporal pole, are supposed to support this operation. The exact role of these areas is nonetheless still debated. In this paper we investigate the hypothesis that different brain regions could be sensitive to different kinds of syntactic computations. We compare the fit of phrase-structure and dependency structure descriptors to activity in brain areas using fMRI. Our results show a division between areas with regard to the type of structure computed, with the left anterior temporal pole and left inferior frontal gyrus favouring dependency structures and left posterior superior temporal gyrus favouring phrase structures.

## INTRODUCTION

When presented with a sentence, all we have is a sequence of visual or auditory stimuli. A key assumption within psycholinguistics is that humans parse this sentence, that is, they construct a syntactic structure that represents the relation between its components. The question we address in this study is whether different brain regions are sensitive to different kinds of syntactic structure building. We compare the predictive power of syntactic structural measures derived from two different families of grammars—dependency grammar and phrase-structure grammar—with regard to the brain activity in language processing areas recorded during naturalistic text listening.

### Brain Areas Underpinning Syntactic Analysis

Sentence processing involves at least two operations: the retrieval of the meaning of single linguistic units from semantic memory (i.e., the mental lexicon), and the computation of the meaning of the structures derived from the combination of these more basic units. This second subprocess likely requires the contribution of some sort of structural analysis, that is, the analysis of the syntactic configuration of the words making up the sentence. In this section we review and motivate a selection of cortical areas that, not uncontroversially, seem to support structural analysis.

The literature reports the involvement of a network of mostly left-lateralised cortical regions including the left inferior frontal gyrus (IFG), the left posterior superior temporal gyrus (pSTG), and the left anterior temporal pole (ATP). There is, however, controversy concerning which brain areas are crucially involved in syntactic processing. A considerable body of literature does report left IFG and left pSTG activation during syntactic processing as opposed to a baseline, usually consisting of random sequences of words (Caramazza & Zurif, 1976; Friederici et al., 2005; Pallier et al., 2011; Snijders et al., 2008; Tyler, Randall, & Stamatakis, 2008; Zaccarella & Friederici, 2015; Zaccarella et al., 2015). However, several other studies do not report activity in left IFG and left pSTG (Bemis & Pylkkänen, 2011; Humphries et al., 2006; Rogalsky & Hickok, 2008), despite using paradigms similar to the above mentioned studies. Moreover, doubts concerning the effective involvement of these areas in syntactic processing are cast by neuropsychological observations. For instance, lesions to the IFG lead to what is clinically known as Broca’s aphasia. These aphasic patients do not perform significantly differently from healthy controls on grammaticality judgments (Linebarger et al., 1983; Wulfeck & Bates, 1991). Similarly, lesion analyses seem to point towards a lack of effect of lesions located in the IFG and pSTG on the performance in basic sentence comprehension (Dronkers et al., 2004; Thothathiri et al., 2012). These studies do not target specific syntactic structures or syntactic structure processing directly. Nonetheless, both tasks—grammaticality judgement and sentence comprehension—are likely to require the computation and the analysis of the syntactic structure of the presented stimuli.

Acknowledging this inconsistency in the literature regarding the involvement of frontal and posterior temporal regions, Matchin et al. (2017) proposed the hypothesis that the left IFG and pSTG may in fact not play a necessary role in syntactic processing. Instead, they claim that these areas are involved only in top-down syntactic prediction, supporting further compositional syntactic operations in the left ATP and the left angular gyrus (AG). The idea that there is a division of labour between the IFG and pSTG, on the one hand, and the ATP and AG on the other was also suggested by an earlier study by Pallier et al. (2011). In this study, Pallier and colleagues analysed the activity recorded during natural language sentence and jabberwocky sentence reading. Searching for brain regions where activation positively correlated with the size of the linguistic constituents, their results isolated a network of left-hemispheric regions that could be dissociated into two major subsets. The left IFG and pSTG showed constituent size effects regardless of whether actual content words were present or were replaced by pseudowords (jabberwocky stimuli). On the other hand, the ATP, the anterior superior temporal sulcus, and the temporo-parietal junction showed constituent size effects only in the presence of lexico-semantic information, suggesting that they may encode sentence-level semantic compositionality.

Besides the above mentioned studies, there is a large literature claiming that the left ATP plays an uncontroversially central role in linguistic processing and has been reported as a central hub for lexical, semantic, and syntactic compositionality. Several studies have pointed to the involvement of the left ATP in processing sentence and phrasal structure. By contrasting the activity recorded during the reading of sentences and of word lists, works such as Mazoyer et al. (1993), Friederici, Meyer, and von Cramon (2000), Humphries et al. (2006, 2007), and Stowe et al. (1998) reported an increase in activity in the ATP for sentence comprehension as compared to word lists. The role of the ATP in processing composition is confirmed by another series of studies which focused on more specific types of syntactic structures. Rather than looking at sentences as a whole, these analyses focused on simple phrasal processing, consisting of the composition of adjectives and nouns (e.g., *red apple*) (Baron & Osherson, 2011; Baron et al., 2010; Bemis & Pylkkänen, 2011; Bemis & Pylkkänen, 2013). These results are confirmed also for a wider range of phrasal and syntactic compositional types and cross-language by Westerlund et al. (2015), and across visual and auditory modality by Bemis and Pylkkänen (2013).

Next to its involvement in syntactic processing, the left ATP is also considered central in semantic memory, a putative subcomponent of long-term memory storing information about the meaning of linguistic units. The first and most compelling proof of this role of the ATP is given by studies on semantic dementia, in which patients showing atrophy of the ATP show a significant impairment in their ability to retrieve and recognise concepts (Hodges, Graham, & Patterson, 1995; Hodges et al., 1992; Mummery et al., 2000; Rogers, Ralph, et al., 2004). This is confirmed also by a large neuroimaging literature (Bright et al., 2005; Gauthier et al., 1997; Moss et al., 2004; Rogers, Hocking, et al., 2006; Tyler et al., 2004). These findings were summarised by Patterson et al. (2007) and led to the formulation of the hub-and-spoke model, which posits that concepts are represented by a network of sensorimotor representations converging in the left ATP, which acts as a hub collecting and controlling modality specific features in order to produce supramodal representations. Following the studies on sentential and phrasal processing, and Patterson’s hub-and-spoke model of semantic memory, it appears that the ATP could play a role in two distinct kinds of composition: one merging words into larger structures (phrases and sentences), and one composing meaning out of more basic semantic features, possibly grounded in sensory-motor representations. This led Westerlund and Pylkkänen (2014) to compare the involvement of the ATP between tasks requiring syntactic and lexical semantic processing, concluding that the two processes might indeed be substantiated by the same cortical mechanism.

### What Form of Syntax?

In the previous section we saw how the debate on the cortical involvement during structural sentence analysis generally points to areas in the left IFG, the left pSTG, and the ATP regions. The question we address in this study is whether they are involved differently in specific syntactic computations.

In this paper we compare *phrase-structure grammars* (PSG) (Borsley, 1998; Chomsky, 1957, 1965) and *dependency grammars* (DG) (Mel’cˇuk, 1988; Kübler et al., 2009; Tesnière, 2015) as two types of structure the brain potentially computes as part of sentence comprehension. The two grammars differ in a number of aspects. DG builds structures solely on the words and on binary relations holding between them, whereas PSG relies on grouping words into phrases that can in turn be grouped into larger phrases, implying a hierarchical structure composed by both surface forms (the words of the sentence) and nonobservable abstract nodes that are assumed to be computed by the human brain. With regard to this study, our aim is not to prove that one grammar is a better formalism than the other. We intend to investigate whether and how the language network in the brain is sensitive to measures derived from both of them. Below we describe in more detail the way these two kinds of grammar differ from each other. To the best of our knowledge, in the field of neurobiology of language, only the present work and the work by Li and Hale (2019) address the two grammars together in the same study. Measures derived from PSG alone have been used in various works (e.g., Brennan et al., 2016; Frank et al., 2012; Nelson et al., 2017).

Inspired by the previous literature we conducted a region of interest (ROI) analysis focusing on the left IFG (pars opercularis, triangularis, and orbitalis separately), the left ATP, and the left STG. We adopted the standard parcellation provided by the commonly used automated anatomical labeling atlas (Tzourio-Mazoyer et al., 2001), which does not divide the STG into subregions. Moreover, the reason for focusing on the IFG subparts is based on Hagoort (2005), which proposes a division of labor inside the IFG depending on the type of binding mechanism performed. We fitted separate linear mixed-effect (LME) models predicting the activity recorded in these areas during naturalistic language listening, using as regressors of interest the structural measures mentioned above. Note that our regressors specify the amount of syntactic processing at each word in our stimuli. These analyses allowed us to identify which area is more sensitive to which type of structural description (PSG or DG). We then conducted a psychophysiological interaction (PPI) analysis investigating how the interaction between each of our ROIs and the rest of the brain is modulated by its preferred structural description from the previous lme-analysis.

Our study is clearly related to the work of Brennan et al. (2016), which showed that activity in the anterior and posterior portions of the left temporal cortex can be predicted using metrics derived from the phrasal structure of the stimuli. Our study is also related to Li and Hale (2019), which followed up on Brennan and colleagues’ study and introduced a measure (structural distance) that combines information from both the phrasal structure and the dependency structure of the stimuli and is intended as a complexity metric quantifying the difficulty implied in memory retrieval. The metric is obtained by counting the number of phrases connecting two words linked by a dependency relation, and it is shown to be able to explain activity in the right anterior and left posterior temporal lobes. Nonetheless, the present study differs from Brennan et al. (2016) and Li and Hale (2019) by separately investigating the effect of dependency and phrasal structure processing in the brain. More specifically, we use measures derived from DG and PSG that are explicitly, and on purpose, kept distinct, under the hypothesis that different parts of the brain might be differentially sensitive to them. Moreover, from a theoretical point of view, Li and Hale’s structural metric is intended as a complexity metric quantifying the difficulty implied in memory retrieval. In contrast, we interpret our metrics instead as correlates of the number of operations necessary to integrate each word into its structural context. Therefore, they are intended as a measure of the effort required by syntactic integration.

### Syntactic Parsing

The assumption presented in the Introduction is that in order to interpret a sentence, the human brain has to establish relations between the words that compose it. For instance, words alone, in isolation, cannot convey the full description of a situation or a state. The following list of words—*paper*, *you*, *this*, and *read*—becomes a suitable description of the action you are performing now only if the relations that the predicate *read* entertains with the subject *you* and the object *this paper* (in turn substantiated by the relation between the determiner *this* and the noun *paper*) are established by your brain. The set of structural relations between the words constitutes the syntactic structure of the sentence. The process that allows you to compute such structure (i.e., to derive relations given a sequence of words) is usually referred to as parsing. The meaning of words, their grammatical category, their relations, and their dependencies with one another determine the interpretation of the sentence they constitute.

### Syntactic Structures

We distinguish between two main approaches to characterize the syntactic structure of a sentence PSGs and DGs. Given a sentence, both grammars produce a hierarchical structure linking or grouping the words in a structure rooted in a governing node (the *root* node). The main difference between the two is that PSG assumes the existence of phrase structures grouping and governing pairs (if the parse tree is binary) of words, whereas DG relies only on word pairings linked by syntactic relations. At a high level of abstraction, what sets a DG structure apart from one derived according to PSG is the fact that DG structures are flatter than PSG structures because they lack phrasal constituents. The structure only consists of the words in a sentence and an associated set of directed binary grammatical relations that hold among them. The only nodes in the DG structure are *terminal nodes* corresponding to surface lexical items as they are encountered by the human reader; no non-terminal, non-observed abstract nodes are introduced. In the following section we expand on the fundamental differences between PSG and DG from both a structural and theoretical point of view.

#### Phrase structure

Phrase-structure grammars define parse structures of sentences as trees composed by terminal and non-terminal nodes. Non-terminal nodes correspond—usually—to phrasal categories as defined by the grammar in use, while terminal nodes (the leaf nodes of the tree) are assigned to the surface forms of the parsed sentence (i.e., its words). Phrase nodes are assigned labels corresponding to syntactic phrasal categories such as noun phrase (NP), verb phrase (VP), adverbial phrase (AP), and determiner phrase (DP).

If the tree is binary (in our definition of a phrase-structure parse we adopt only binarized trees) phrasal nodes can have a maximum of two child nodes that can be either other phrasal nodes or leaf nodes (words). A parent node can only consist of a phrasal node; it is also referred as non-terminal. Words can only be children of non-terminal phrasal nodes and are referred to as terminal or leaf nodes because they are not hierarchically higher than any other node. Besides phrasal and leaf nodes, the phrase-structure parse also contains a root node. A root node is a node that is not a child of any other node. Given a parse of a sentence, the resulting tree contains only one root node. The root note corresponds to the category S, governing the sentence as a whole.

(1)  The man saw a brown dog in the park.

**Figure 1.**
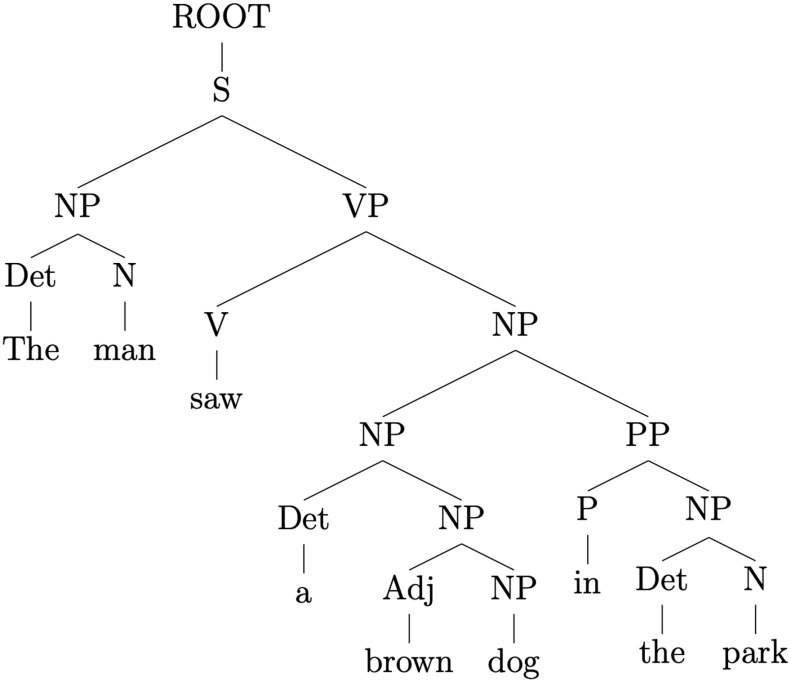
Phrase-structure parse of Sentence 1.

As an example, as displayed in Figure 1, the parse of Sentence 1 contains eight labeled phrase structures, including S, and constitutes a nested binary-branching tree. The words of the sentence (*the*, *man*, *saw*, *a*, *brown*, *dog*, *in*, *the*, and *park*) correspond to the terminal nodes. Following the structure of the parse tree in a top-down fashion: S branches into NP and VP, respectively. The left-hand child (NP) is composed of a determiner leaf node *the* and a noun *man*; whereas the right-hand child of S (VP) has in turn as left-hand child a terminal node (the finite verb *saw*) and as its right-hand side child another noun phrase (NP). This last NP branches into another NP and a prepositional phrase (PP). These two last phrases both split into a left-hand terminal child (respectively *a* and *in*) and into NP as a right-hand child. The latter two are both composed of terminal nodes (*brown*, *dog*, *the*, and *park*).

#### Dependency structure

Dependency grammar describes a sentence as a set of relations between pairs of words—a head and a dependent—composing it. The relations can be called dependencies and correspond to grammatical functions. The relations, and the words they link, are the only elements composing the structure (Kübler et al., 2009; Mel’cuk, 1988; Tesnière, 2015). In a dependency structure the finite verb is often taken to be the structural hub of the sentence. All other words are either directly or indirectly connected to the verb by dependencies.

**Figure 2.**
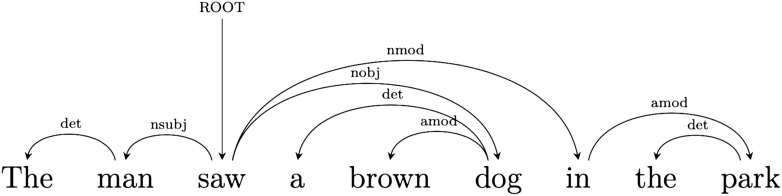
Dependency parse of Sentence 1.

Take for instance Sentence 1 above. The dependency graph in Figure 2 represents the dependency structure of the sentence in terms of typified head-dependent relations: The main verb (*saw*) acts as head for *man* and *dog*, with which the verb is in a *subject* and an *object* relation respectively. A dependent of one dependency relation can in turn be the head of another. For instance *dog* is head of *brown*, with which it is linked by a *modifier* relation, and also head of article *a* via a *determiner* relation. Dependencies can be instantiated between words far apart in the sequential structure of the sentence.

**Figure 3.**
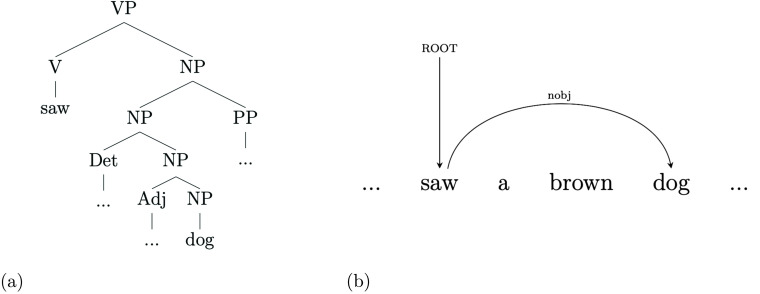
Comparison between the structures mediating the relation between the sentence’s main verb (*saw*) and its object (*dog*) in the phrase-structure (a) and dependency structure (b) parses of Sentence 1.

Relations that hold between words are captured in structurally different manners by dependency structure and phrase-structure parses of the same sentence. Take for instance the relation between *saw* and *dog*, respectively the main verb and the direct object in Sentence 1. As it is apparent from the graph path between these two items in Figure 3, DS directly captures their predicate-object relation by means of a simple directed edge (Figure 3b), whereas PS relies on three intervening noun phrases and a governing verb phrase (Figure 3a).

### The Relation Between Dependency and Phrase-Structure Grammars

Dependency grammar and phrase-structure grammar are two different syntactic formalisms, using different structural primitives (dependency relations and phrases). There has been some discussion in the field of theoretical linguistics with regard to whether they capture the same information or to what degree the structures they sanction are equivalent (Hays, 1964; Jung, 1998).

Discussing the linguistic information the two grammars capture, Rambow (2010) notes that, from a theoretical linguistic point of view, dependency and phrase-structure describe distinct syntactic entities, and thus are not strictly equivalent. Dependencies capture direct relations between words, identical to thematic functions such as subject, object, modifier, etc. Syntactic phrase-structure, on the other hand, is not so much about functional relations between words, but about the recursive grouping of sentence constituents (words and phrases), such that at each level, each grouping acts as a syntactic unit (Schneider, 1998). Moreover, according to Jung (1998) only dependencies can express the syntactic word-to-word relations of a sentence, but only constituency can express the linear order of a sentence. Jung, therefore, sees the two grammars as complementary and not equivalent.

Following these last observations, we consider dependency and phrase-structure distinct, and the type of information that they capture as nonequivalent.

### Hypotheses Concerning Brain Areas Involvement

Li and Hale (2019) showed that after controlling for structural distance (which combines both phrasal and dependency information), phrase structural predictors do not significantly contribute to the explanation of activity in the anterior temporal lobe. We hypothesize that predictors solely based on DG might significantly predict activity in the anterior temporal lobe. This intuition is also grounded on the results reported by Westerlund et al. (2015), which showed modulation of ATP activity as a function of the type of syntactic relation entertained by pairs of words (e.g., adjective-noun, adverb-verb). Word pairs, entertaining relations comparable to dependency relations, elicited larger activation in the left ATP compared to random pairings of words, suggesting that this area might play a role in syntactic processing in lines with DG.

## MATERIALS AND METHODS

### Participants and Stimuli

We re-analysed data from an fMRI study on language comprehension of auditory presented narrative texts (Lopopolo, Frank, van den Bosch, Nijhof, & Willems, 2018). Here we briefly present the data collection procedure, preprocessing, and stimuli employed. Full details can be found in the original articles (Lopopolo, Frank, van den Bosch, Nijhof, & Willems, 2018; Willems et al., 2016). (The dataset is available at https://osf.io/utpdy/.)

Twenty-four healthy, native speakers of Dutch (8 males; mean age 22.9, range 18–31), without psychiatric or neurological problems, with normal or corrected-to-normal vision, and without hearing problems, took part in the experiment. All participants except one were right-handed by self-report, and all participants were naive with respect to the purpose of the experiment. Written informed consent was obtained in accordance with the Declaration of Helsinki, and the study was approved by the local ethics committee (Central Committee on Research Involving Human Subjects, CMO region Arnhem–Nijmegen, The Netherlands, protocol number 2001/095). Participants were paid either in money or in course credit at the end of the study.

Stimuli consisted of three excerpts from three distinct literary novels extracted from the Spoken Dutch Corpus (Corpus Gesproken Nederlands [CGN]; (Oostdijk, 2000). We used the audio recordings of these texts, and no other data or metadata from the CGN was used for our analyses. The excerpts were spoken at a normal rate, in a quiet room, by female speakers (one speaker per story). Stimulus durations were 3:49 min (622 words), 7:50 min (1,291 words), and 7:48 min (1,131 words). Reversed speech versions of the stories were created with Audacity 2.03 (https://www.audacityteam.org/). These were used as a low-level baseline in the analysis.

### Procedure

Participants passively listened to the three narratives and their reversed versions (for a total of six runs) inside the MRI scanner. Each story and its reversed speech counterpart were presented following each other. Half the participants started with a non-reversed stimulus, and half with a reversed speech stimulus. Participants were instructed to listen to the materials attentively, which in practice is only possible for the three narratives, and not for the reversed speech counterparts. There was a short break after each fragment.

Stimuli were presented with Presentation 16.2 (https://www.neurobs.com/). Auditory stimuli were presented through MRI-compatible earphones. In order to make sure participants could correctly perceive the stimuli, the actual experimental sessions were preceded by an in-scanner volume test, in which a fragment from another story with a comparable voice and sound quality was presented and the volume was adjusted to the optimal level based on feedback from the participant.

After the scanning session, participants were tested for their memory and comprehension of the stories. The participants were not informed in advance about the test in order to avoid attentional biases during the passive listening to the stories.

### FMRI Data Acquisition and Preprocessing

Images of blood oxygen level-dependent (BOLD) changes were acquired on a 3-T Siemens Magnetom Trio scanner (Erlangen, Germany) with a 32-channel head coil. Pillows and tape were used to minimize participants’ head movement, and the earphones that were used for presenting the stories reduced scanner noise. Functional images were acquired using a fast T2*-weighted 3D echo planar imaging sequence (Poser et al., 2010), with high temporal resolution (time to repetition: 880 ms, time to echo: 28 ms, flip angle: 14°, voxel size: 3.5 × 3.5 × 3.5 mm, 36 slices). High resolution (1 × 1 × 1.25 mm) structural (anatomical) images were acquired using a T1 sequence.

Preprocessing was performed using SPM8 (https://www.fil.ion.ucl.ac.uk/spm) and MATLAB 2010b (https://www.mathworks.nl/). The first four volumes were removed to control for T1 equilibration effects. Rigid body registration was used to realign images. Images were realigned to the first image within each run. The mean of the motion-corrected images was then brought into the same space as the individual participant’s anatomical scan. The anatomical and functional scans were spatially normalized to the standard MNI template, and functional images were re-sampled to 2 × 2 × 2 mm voxel sizes. Finally, an isotropic 8 mm full-width at half-maximum Gaussian kernel was used to spatially smooth the motion-corrected and normalized data.

### Syntactic Measures

Both dependency and phrase-structure parses of the sentences composing the stimulus texts were derived using a computational *parser* developed and trained for Dutch (ALPINO; van Noord, 2006). ALPINO has been profusely used in several studies requiring syntactic analyses of Dutch linguistic material, ranging from natural language processing to psycholinguistic and neurolinguistic studies. We have expanded the section describing the parser with a more comprehensive review of previous neurolinguistic and psycholinguistic studies using this parser to derive measures from phrase-structure and dependency grammars. More specifically, Bastiaanse et al. (2009) used ALPINO to generate dependency structures from corpus data used to investigate the effects of frequency and complexity on agrammatic production. Similarly, Lopopolo, Frank, van den Bosch, and Willems (2019) used ALPINO to study the relationship between dependency structures and patterns of eye movement during reading, demonstrating the validity of ALPINO as a tool for DG-based structural analyses. On the other hand, Brouwer et al., 2010 used ALPINO to obtain PSG representations in their study of Dutch syntactically ambiguous structures. Moreover, studies conducted by Kos and colleagues (Kos, van den Brink, & Hagoort, 2012; Kos, van den Brink, Snijders, et al., 2012; Kos, Vosse, van den Brink, & Hagoort, 2010) made extensive use of the Dutch treebank (CLEF corpus), which was obtained by using ALPINO (van der Beek et al., 2002).

The grammar implemented by ALPINO grammar is a wide-coverage head-driven phrase structure grammar. Nonetheless it has been augmented to enable it to output dependency structures compatible with our definition of DG and based on the guidelines of the CGN (Oostdijk, 2000).

The output of ALPINO is able to return sentence parses consistent with the principles of PSG, as well as DG. Moreover, it is able to generate both these two types of parses from within the same framework, making it convenient and allowing us to avoid inconsistencies derived from using different parsers built and trained on different data. From each of these parse structures we derive a measure approximating the operations performed in order to integrate each word into the syntactic structure computed at the point of its presentation. The next sections will describe these measures in detail.

#### Dependency parse

In order to describe the dependency structure of a sentence, the ALPINO parser creates a structure composed by dependency triples consisting of a head word, the type of dependency relation, and its dependent word. A parse is produced for each sentence independently; therefore, no relation can be assigned between words belonging to different sentences.

In order to describe the operation required to integrate a word at a time into the incrementally built dependency structure of the sentence we adopted the number of left-hand side relations entertained by each word. As described above, every word in a sentence entertains at least one relation with another word in the same sentence. Every non-final and non-initial word can have relations with a variable number of other words on its right and its left. Logically, a sentence-initial word can only have relations with words to its right, and a sentence-final word can only be linked to words on its left. In order to quantify the operations required to integrate a word *w* into the structure constructed up to its presentation, only relations with a head and possible dependents on the left-hand side of *w* are counted. In other words, from the dependency structure of a sentence, we count the number of left hand-side edges for each word *w* in the sentence (dependency structure left relations, or DSlrels, see Table 1).

**Table 1.** Number of left-hand dependency relations (DSlrels) per word *w* in the example Sentence 1

	The	man	saw	a	brown	dog	in	the	park
**DSlrels**	0	1	1	0	0	3	1	0	1

For example, the word *dog* in the sentence has two dependent relations with two words to its left (*a* and *brown*), no dependents to its right, but one head to its left (*saw*). The word *park*, being sentence-final, does not have any links on its right, but it has one head (*in*) and one dependent (*the*) to its left. From a neurobiological point of view, the assumption is that all dependency relations have equal cost. Under our hypothesis, each relation engenders an equivalent BOLD response independently from its type and the distance between head and dependents.

#### Phrase-structure parse

As for the phrase-structure parse, the texts of the three stories presented to the participants were fed to the ALPINO toolbox for Dutch natural language processing in order to generate this time a phrase-structure parse for each sentence (van Noord, 2006).

In order to quantify the number of syntactic operations per word required to construct a phrase-structure parse of the input sentence, we measured the number of closed phrase structures allowed after the introduction of each novel word (PSxps). Such a measure is computed by considering whether a word or phrase is a right-hand or left-hand side child of its parent phrasal node. In case the word in question is the right-hand side child, the parent phrasal node is considered complete and therefore closed. This proceeds recursively, evaluating whether a closed phrasal node is in its turn the right-hand side child of a higher order parent phrasal node, allowing it to be closed. For instance, according to the phrase-structure parse of Sentence 1 (Figure 1), the first instance of the word *The* is the left-hand side child of an NP structure; for this reason this NP is not complete and cannot be closed. Therefore, the value of PSxps for *The* is 0. On the other hand, *man* is the right-hand side child of the same NP and therefore this phrase structure can be closed at this word position, allowing the assignment of value 1 to *man*. Following the same reasoning, *dog* is the left-hand side child of another NP allowing for its closure. This last NP is in turn the left-hand side of a higher NP structure. Therefore the word *dog* is assigned value 2 because its presentation allows for the completion of two nested phrase structures. Table 2 contains the PSxps values for the whole Sentence 1.

**Table 2.** Number of closed phrase structures (PSxps) per each word *w* in the example Sentence 1

	The	man	saw	a	brown	dog	in	the	park
**PSxps**	0	1	0	0	0	2	0	0	5

This measure is computed under the following simplifying assumptions: that phrase-structure trees are binary (i.e., as explained above, that they can have only two children), and that parsing proceeds incrementally left-to-right. Similarly to what was stated for dependency relations above, the assumption is that all the integration of phrase structures have equal cost. Under this assumption, each phrase engenders an equivalent BOLD response independently from its type (NP, VP, etc.) and distance from the root node.

### Controlling for Lexical Frequency and Word Surprisal

To control for other factors known to influence brain activation during language comprehension, we added log-transformed lexical frequency and surprisal as covariates to the analysis (Lopopolo, Frank, van den Bosch, & Willems, 2017; Willems et al., 2016). Log2-transformed lexical frequency per word was computed using the SUBTLEX NL corpus (Keuleers et al., 2010). Surprisal was computed from a second-order Markov model, also known as a trigram model, trained on a random selection of 10 million sentences (comprising 197 million word tokens; 2.1 million types) from the Dutch Corpus of Web (NLCOW2012; Schäfer & Bildhauer, 2012). Surprisal of word *w*_*t*_ is the negative logarithm of the conditional probability of encountering *w*_*t*_ after having read sequence *w*_*t*−2_, *w*_*t*−1_, or −log *P*(*w*_*t*_|*w*_*t*−2_, *w*_*t*−1_). The computation was performed by the SRILM toolbox (Stolcke, 2002).

### Analyses

Our main analysis consists of ROI-wise linear model fitting using as predictors the syntactic structure measures described in the previous sections, together with lexical frequency and surprisal as regressors of no interest.

Besides ROI analyses, we also conducted whole-brain PPI analyses and whole-brain general linear model analyses. The former was performed in order to test the interaction between the regions of the language network and the rest of the brain with regard to the type of syntactic structure considered in this study. The latter was instead performed in order to have a wider—less biased—view of the possible division of labour between dependency and phrase-structure parsing in the brain.

### ROI Analyses

We chose six separate left-hemisphere anatomical ROIs to selectively test the contribution of our two syntactic measures as predictors of BOLD activity. These regions were the STG (including Wernicke’s area), the middle temporal pole (mATP), the superior temporal pole (sATP), the IFG pars opercularis (IFG_oper), the IFG pars triangularis (IFG_tri), and the IFG pars orbitalis (IFG_orb). The regions are defined following the automated anatomical labeling (AAL) atlas (Tzourio-Mazoyer et al., 2001) as implemented in SPM12. We then computed the average BOLD signal for each of our 24 participants and six ROIs.

For each of the six ROIs, we fitted three LME models predicting the average BOLD signal. The first model (Base, 2 below) contains as predictors only probabilistic information (lexical frequency and surprisal) relative to each word. Estimates from the motion-correction algorithm (three rotations and three translations per run) were additionally added as regressors of no interest. In order to assess the effect of dependency and phrase-structure measures to each ROI’s BOLD signal, models 3 and 4 were fitted with one of our syntactic measures (DSlrels, PSxps) each in addition to the same covariates of the Base model. In addition, we included by-subject random intercepts, as well as the by-subject random slopes for surprisal and log-transformed word frequency.

(2)  Base model: *BOLD* 1 + *lexf req* + *surprisal* + *m*1 + *m*2 + *m*3 + *m*4 + *m*5 + *m*6 + (1|*subject*) + (1 + *surprisal*|*subject*) + (1 + *lexf req*|*subject*)
(3)  DSlrels model: *BOLD* 1 + *lexf req* + *surprisal* + *DSlrels* + *m*1 + *m*2 + *m*3 + *m*4 + *m*5 + *m*6 + (1|*subject*) + (1 + *surprisal*|*subject*) + (1 + *lexf req*|*subject*)
(4)  PSxps model: *BOLD* 1 + *lexf req* + *surprisal* + *P Sxps* + *m*1 + *m*2 + *m*3 + *m*4 + *m*5 + *m*6 + (1|*subject*) + (1 + *surprisal*|*subject*) + (1 + *lexf req*|*subject*)

We compared the syntactic models (DSlrels and PSxps) against the Base model in order to test whether the introduction of the syntactic measure significantly improved the fit to the data. We also directly compared the DSlrels and the PSxps models in order to test for specific syntactic structure selectivity in our six ROIs. Model comparisons were performed using the likelihood-ratio test. The models were fit by maximum likelihood.

### PPI Analysis

The ROI analysis introduced above was aimed at determining the contribution of our structural measures to the activity of left inferior frontal, superior temporal, and antero-temporal regions of the brain—areas that are claimed to be responsible for structural analysis of linguistic stimuli. In order to investigate the interplay between these (and other) brain regions, we additionally investigated PPIs. PPI is a functional brain connectivity analysis method, developed to estimate context-dependent changes in functional connectivity cortical areas (Friston, 2011; Friston, Büchel, et al., 1997). It models the way brain activity is determined by the activity of a preselected seed region when modulated by experimental conditions or parameters (modulator). The analysis takes the activity of the seed region (the *physiological* component) and a modulator (the *psychological* component) and fits a voxel-wise linear model using as predictor of interest the product of these two components (the *psychophysiological* interaction). In this way the PPI identifies brain regions whose activity depends on an interaction between psychological context (the task) and physiological state (the time course of brain activity) of the seed region (O’Reilly et al., 2012).

The activity of each seed region was computed by fitting a general linear model containing as predictors, our structural measures, and as covariates, lexical frequency and surprisal and parametric head movement. The eigenvalue of the voxels inside the ROI showing suprathreshold activation for the regressor of interest was used to compute the physiological component of the PPI. This was conducted at single-subject level with a significance level of *p* < 0.05. The regressor of interest used for ROI-wise voxel selection acted also as the psychological modulator for the subsequent PPI analyses proper, which consisted of fitting another subject-level whole-brain general linear model using as regressor of interest the product of the seed activity and the modulator measures, and as covariates the seed activity and modulator themselves. The goal was to then identify those voxels (both at single subject and group level) that respond significantly to the interaction between seed activity and modulator.

### Whole-Brain Analysis

At the single-subject level, the observed BOLD time course in each voxel was subjected to a regression analysis, testing for voxels in which the covariates of interest (DSlrels, PSxps) explain a significant proportion of variance of that voxel time course (Friston, Holmes, et al., 1995). Before the actual analysis, one regressor modelling the duration of each single word was created for each story. This regressor was convolved with the hemodynamic response function to account for the delay in BOLD activation respective to stimulus presentation. Besides the covariates of interest, log-transformed lexical frequency per word—computed using the SUBTLEX-NL corpus (Keuleers et al., 2010)—and per-word surprisal were introduced. They were used as regressor of no interest to statistically factor out effects of stochastic properties of the words. The estimates from the motion correction algorithm (three rotations and three translations per run) were additionally added as regressors of no interest.

We were interested in assessing which voxels are more sensitive to DSlrels as compared to PSxps and vice versa. In order to do so, we contrasted these two regressors of interest in order to identify voxels that are selective for each one of the regressors over and above the contribution of the other (DSlrels > PSxps, PSxps > DSlrels). The significance of these contrasts was assessed by computing the *t* statistic over participants of this difference score for each voxel in the brain. The resulting multiple comparison problem was solved by means of combining a *p* < 0*.*05 voxel threshold with a cluster extent threshold determined by means of 1,000 Monte Carlo simulations, after estimation of the smoothness of the data (Slotnick et al., 2003) applied for each separate contrast both for the single and the total models. Clusters of a size exceeding the number of voxel thresholds corresponded to statistically significant effects (*p* < 0.05 level, corrected for multiple comparisons).

## RESULTS

### ROI Analyses

#### Comparison against the Base model

**Figure 4.**
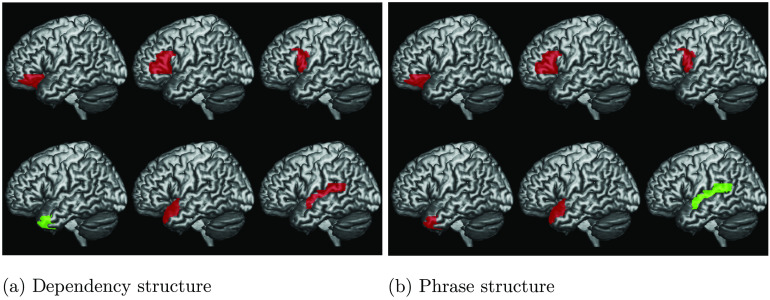
Cortical illustration of the likelihood ratio test between the Base model and the DSlrels model (a), and between the Base model and PSxps model (b). Green indicates ROIs where the likelihood ratio test returns significant results after Bonferroni correction (adjusted alpha = 0.002, 0.05/18, 6 ROIs and 3 models). Red indicates ROIs for which the syntactic measure does not significantly improve fit. Note the different selectivity between anterior and posterior temporal areas of the network with regard to the type of syntactic structure.

We computed the likelihood-ratio test for the difference in fit between the Base model and each of the two syntactic models above across the six ROIs. This allowed us to test whether the introduction of syntactic measures significantly improves the fit of the LME model to the BOLD signal. The results of these analyses are reported in Table 3 and Figure 4.

**Table 3.** Likelihood ratio test between the Base model and each of the two models fitted with one of two syntactic measures derived either from dependency and phrase-structure parses

**ROI**	**DSlrels**		**PSxps**	
	*χ* ^2^	***p***	*χ* ^2^	***p***
STG	1.1009	0.2941	**41.8453**	**0.0000** *
mATP	**12.7514**	**0.0003** *	0.7733	0.3792
sATP	2.0497	0.1522	0.2541	0.6142
IFG_oper	3.8301	0.0500	0.8991	0.3430
IFG_tri	2.9034	0.0884	0.3062	0.5800
IFG_orb	0.7947	0.3727	0.3617	0.5476

*Note.* *p < 0.002, Bonferroni-adjusted alpha 0.05/18, 6 ROIs and 3 models.

Tests were conducted using Bonferroni adjusted alpha levels of 0.002 per test (0.05/18, 6 ROIs, and 3 models). The results indicate that there was a strong effect of PSxps in the left STG (41.8453, *p* = 0.0000). On the other hand, the DG structure building measure—DSlrels—had a strong effect in the left middle TP (12.7514, *p* = 0.0003). A weaker effect of DSlrels was also present on the left IFG pars opercularis (3.8301, *p* = 0.0500), but not significant after Bonferroni correction.

#### Comparison between models

Table 4 contains the results of the log-likelihood test between the DSlrels and PSxps models. Syntactic operation measures of DSlrels and PSxps were directly compared to assess the prominence of one or the other as predictor of activity inside our ROI pool.

**Table 4.** Likelihood ratio test between dependency relations and phrase structures

**Model comparison**	**ROI**	*χ* ^2^	***p***
PSxps > Dslrels	STG	**40.7176**	**0.0009** *
DSlrels > PSxps	mATP	**11.9757**	**0.0009** *
	IFG_oper	2.9306	0.0430
	IFG_tri	2.5970	0.0540

*Note.* *p < 0.004, Bonferroni-adjusted alpha 0.05/12, 6 ROIs and 2 models.

Tests were conducted using Bonferroni adjusted alpha levels of 0.004 per test (0.05/12, 6 ROIs, and 2 models). The left STG confirms a strong preference for the phrase-structure measure, with the model fitted with PSxps significantly outperforming the model fitted with DSlrels. DSlrels instead outperforms its PSG counterpart in the middle ATP and in the pars opercularis and triangularis of the IFG, yet not surviving Bonferroni correction.

### PPI Analyses

The results presented in the previous section highlight a preferential selectivity for dependency structure in the left ATP and IFG, and a selectivity for phrase structure in the left STG.

**Figure 5.**
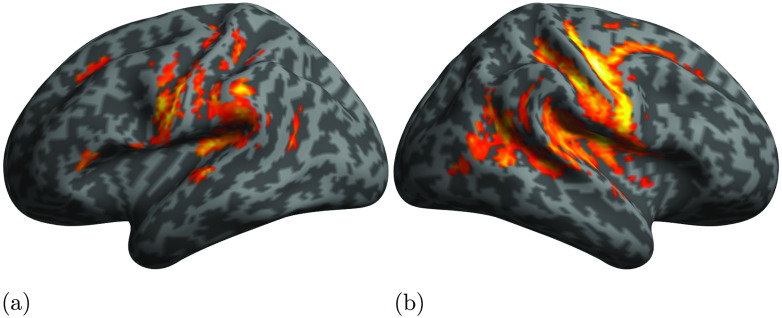
Results of the PPI analyses using as seed the left STG and as modulator PSxps.

**Figure 6.**
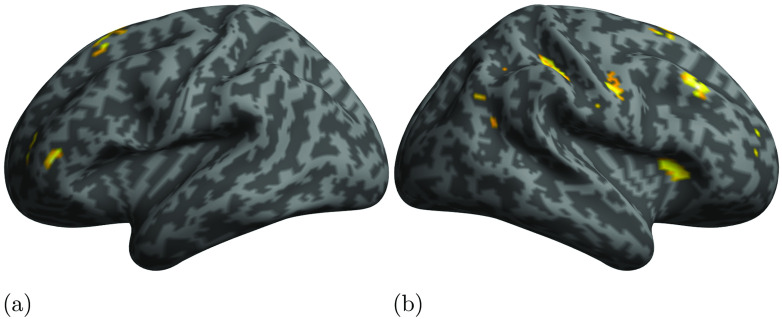
Results of the PPI analyses using as seed the left ATP and as modulator DSlrels.

**Figure 7.**
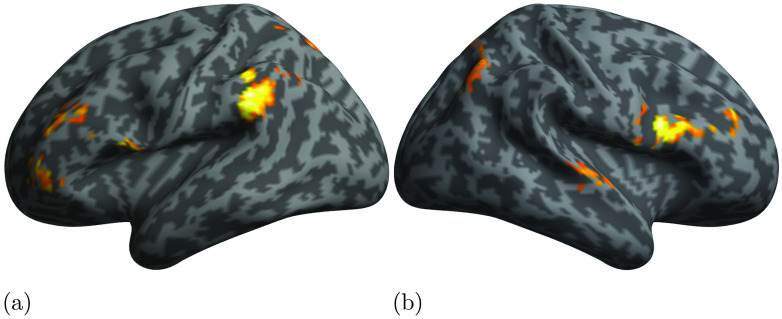
Results of the PPI analyses using as seed the left IFG and as modulator DSlrels.

In this section we present the results of PPI analyses aimed at assessing the relation between activity in our ROIs as modulated by the processing of either phrase structure or dependency structure. Since the STG showed selectivity for phrase structures, and the IFG and ATP for dependency structures, we conducted three separate whole-brain PPI analyses. We first checked for brain areas in which the activity was driven by STG activity (physiological seed) modulated by PSxps structural measure (psychological modulator). We then used the activity of either IFG or ATP as physiological seeds, and DSlrels as modulator in order to assess the contribution of these areas and structure to the activity of the rest of the brain during language processing. Table 5 and Figure 5 report the results of the PPI analyses using STG as physiological seed and PSxps as activity modulator. The results highlighted large clusters in the bilateral central sulci (CS) and precentral gyri (PCG) encompassing both bilateral primary motor and premotor cortices. Activation was also observed for the bilateral posterior temporal and perisylvian cortices. Interestingly, activity in the left IFG was also driven by the interaction between the activity in the left STG and the PSxps measure. Tables 6 and 7, and Figures 6 and 7 report the results of using DSlrels as modulator and ATP and IFG as physiological seeds respectively. These results indicate that the activity of the left ATP, modulated by DSlrels, explains the activity in a limited set of clusters located in the bilateral prefrontal cortex (PFC).

**Table 5.** Results of the PPI analyses using as seed the left STG and as modulator PSxps

**Area**	**MNI coord.**	**T**	***p***	**Cluster size**
left IFG (triangularis)	−50 22 0	3.08	0.003	94
left PCG (premotor)	−58 −4 28	3.16	0.002	2922
left MTG (auditory)	−34 −30 10	3.12	0.003	
left CS (primary motor)	−54 −12 36	3.00	0.003	
right CS (primary motor)	36 −14 38	4.20	*p* < 0.001	10896
right PCG (premotor)	36 −12 45	3.62	*p* < 0.001	

**Table 6.** Results of the PPI analyses using as seed the left ATP and as modulator DSlrels

**Area**	**MNI coord.**	**T**	***p***	**Cluster size**
left caudate	−24 0 20	3.27	0.002	3640
right dorsolateral PFC	44 26 40	3.34	0.002	142

**Table 7.** Results of the PPI analyses using as seed the left IFG and as modulator DSlrels

**Area**	**MNI coord.**	**T**	***p***	**Cluster size**
left IFG (triangularis)	−58 16 6	3.64	*p* < 0.001	100
left SMG	−54 −40 34	3.71	*p* < 0.001	625
left AG	−64 −42 26	3.18	0.002	
left anterior PFC	−44 44 20	3.36	*p* < 0.001	484
right anterior PFC	44 44 22	3.72	*p* < 0.001	1014
right SPG	30 −66 62	3.29	0.002	512

Activity in the left IFG modulated by DSlrels explains the activity in the left supramarginal gyrus (SMG) and AG in the posterior perisylvian cortex. Activity in the bilateral anterior PFC is also driven by this interaction, as is part of the middle banks of the right STG.

### Whole-Brain Analyses

Tables 8 and 9 contain the results of the whole-brain analysis contrasting PSG and DG measures.

**Table 8.** Whole-brain results areas that are more sensitive to PSxps as compared to DSlrels (PSxps > DSlrels)

**Area**	**MNI coord.**	**T**	***p***	**Cluster size**
left hippocampus	−32 −14 −18	5.32	*p* < 0.001	701
left MTG	−34 8 −22	3.36	*p* < 0.001	
left fusiform	−52 −42 −14	4.14	*p* < 0.001	83
left occipital	−12 −90 −10	3.88	*p* < 0.001	1637
left AG	−30 −72 38	3.78	*p* < 0.001	
left IFG (orbitalis)	−20 30 −10	3.58	*p* < 0.001	198
right occipital	42 −80 8	3.48	*p* < 0.001	2046

**Table 9.** Whole-brain results areas that are more sensitive to DSlrels as compared to PSxps (DSlrels > PSxps)

**Area**	**MNI coord.**	**T**	***p***	**Cluster size**
left AG	−40 −58 22	5.00	*p* < 0.001	9207
right caudate	6 14 8	4.56	*p* < 0.001	
left SFG	−12 52 26	3.12	0.002	216
right posterior CC	12 −22 38	3.32	*p* < 0.001	139

Contrasting PSxps and DSlrels (PSxps > DSlrels, 8) highlights the role of the IFG (orbitalis), AG, fusiform, and hippocampus in the left hemisphere, and of the bilateral occipital cortex.

On the other hand, contrasting DSlrels and PSxps in the other direction (Dslrels > PSxps, 9) indicates an involvement of the left superior frontal gyrus (SFG) and the right caudate.

## DISCUSSION

The goal of our experiment was to investigate whether parts of the brain that have previously been implicated in syntax are sensitive to different types of syntactic operations involved in parsing sentences. We investigated whether brain activity of six left hemispheric regions was better explained by DG or by PSG. The two grammars were not meant to represent contrasting hypotheses, and our aim was not to prove that one is a better formalism than the other. Our results suggest that both grammars can explain variance in cortical areas supposedly involved in syntactic processing, and that they appear to do so for different areas of the brain.

These results partially differ from those reported by Li and Hale (2019). Their structural distance metric explains activity in the right anterior and left posterior temporal cortex. Considering that the PSxps instead explained activation in the left STG, whereas the DSlrels explained activation in the left ATP, their observation that structural distance shows activation in both anterior and posterior superior temporal areas might be due to the fact that it implicitly contains information conflating the dependency and phrasal structure of the stimuli. We believe that these differences are caused by a different use of information from dependency and phrase-structure grammars in the two studies. On the other hand, Li and Hale’s context-free grammar-based measure (comparable to our PSxps) does explain activity in the left posterior temporal lobe, which we believe is in line with our results showing the PSxps explaining activity in the left STG.

### A Syntactic Division of Labour

The results of a series of ROI analyses show that dependency structure measures significantly explain activity in the left ATP, and that phrase-structure measures seem instead to explain activity in the left STG. These results overall seem to point towards a general division of labour between anterior temporal areas (responsible for the computation of dependency representations of the sentence) and more posterior temporal areas involved, instead, in phrase-structure analysis.

### The Role of the Anterior Temporal Lobe

Our results indicate a relation between ATP activity and the number of left-hand side dependency relations at the word level, which was adopted as a quantification of the operation the human brain is supposed to carry out in order to integrate each word into the dependency parse of the sentence that it is embedded in. This therefore seems to indicate that the ATP acts as a combinatorial hub that binds together words according to relations similar to the ones characterizing a dependency parse. These results are in line with previous studies that describe this area as a hub for composition during sentence comprehension (Friederici et al., 2000; Humphries et al., 2006, 2007; Mazoyer et al., 1993; Stowe et al., 1998).

Dependency structure, composed by binary typified relations, might also be close to the two-word stimuli that were found to elicit activation in the ATP by Baron et al. (2010) and by Westerlund et al. (2015). Baron et al. (2010) observed a modulation of activity in this region when adjective–noun pairs were presented. The relations of adjectives and nouns are directly captured by the modification relations in the type of dependency parses we adopted in this study. Similarly, an interesting parallelism between the typified relations that constitute dependency graphs and the types of two-word stimuli presented in Westerlund et al. (2015) might help in understanding why dependency structure might be a correct way of characterizing the type of job performed by the ATP. Dependency relations directly link pairs of words according to the type of role they play in a sentential–semantic relationship. They can be grouped—broadly speaking—in verb-argument relations (i.e., the relations established between a predicate and its subject, object, or complement, or modifier relations).

Modifier-type dependency relations can be exemplified by the link between an adjective and a noun, an adverb and a verb, a determiner and a noun, and so on. Westerlund et al. (2015) demonstrated that a wide range of “composition modes” affect the activity of the left ATP. These modes consist of two-word sequences classified either as modification (Adjective-Noun Adverb-Verb Adverb-Adjective) or argument saturation (Verb-Noun Preposition-Noun Determiner-Noun). These modes (aside from the fact that they involve word pairs) resemble the classification of the dependency relation types. Therefore it seems natural to suggest that, on the basis of our results and the results found in the literature, DG offers a reasonable formalization of the type of structure employed (or constructed) in the left ATP.

Our results therefore confirm that the left ATP can serve as the locus where sentence-level semantic representations are computed, and that these representations might be produced by combining the sentence constituent words following the structure specified in a fashion comparable to a dependency parse.

### The Role of the Left IFG

The ROI analyses show a small effect of dependency structure measures on the activity in the left IFG (pars opercularis). This area plays center stage in several studies on language and syntactic processing and it is often associated with the activity in the left pSTG (Caramazza & Zurif, 1976; Friederici et al., 2005; Pallier et al., 2011; Snijders et al., 2008; Tyler et al., 2008; Zaccarella & Friederici, 2015; Zaccarella et al., 2015). Our results seem to indicate that this area might work in concert with the left ATP in building sentence-level representations that follow the structure described by word-word dependency relations. This is potentially compatible with the memory, unification, and control framework (Hagoort, 2013) that predicates a role for the IFG in integrating words into their sentential and discourse context.

Besides the ROI analyses, the PPI and whole-brain analyses provide a somewhat more complex picture. Activity in part of the left pars triangularis is explained by the activity of the left STG modulated by the phrase structure, whereas activity in another portion of the same subregion is linked to the activity in the pars opercularis modulated by the dependency structure. The whole-brain analyses instead indicate an involvement of the left pars orbitalis in phrase-structure processing.

In light of these observations, it is possible that different subregions of the left IFG support the analysis of different syntactic structures, in concert with either the left ATP or the left STG. In particular, the pars opercularis might work in concert with the left ATP in building sentence-level dependency representations, whereas the pars orbitalis performs operations related to the ones carried on in the left STG, having to do with hierarchical phrasal representations of the sentence. The pars triangularis, in different ways linked to the computation of dependency structure in the ATP and the phrasal analysis in the STG, might—it is very tentative to say—act as a buffer between these two areas and their syntactic operations.

These explanations are still at the level of speculation, and we defer to further investigations before drawing any stronger conclusions.

### Additional Areas Involved in Dependency Parsing

Whole-brain analyses highlighted an additional set of regions that are more sensitive to dependency structure as compared to phrase-structure measures. In addition to the ROI results, we observed an involvement of other brain structures: the left AG, the right posterior cingulate cortex, and the left superior frontal gyrus.

These patterns of activation might indicate that dependency structures correlate with working memory mechanisms subserving syntactic parsing. The PFC has been mentioned as a central player in working memory studies, including in the domain of language processing and sentence comprehension (D’Esposito & Postle, 2007; Nee & D’Esposito, 2016). In addition, Bonhage et al. (2014) reported the involvement of also the inferior parietal cortex (including the AG) and areas bordering the cingulate cortex and the precuneus during the encoding in working memory of short sentence fragments (4 or 6 words).

One may argue that the number of DSlrels governing each word in the stimulus is simply modeling the load on working memory resources required for word-by-word sentence processing. In other words, given a sentence, the brain has to store in memory each word incrementally until the recipient of a dependency relation with each word is presented—and eventually integrated. In this sense, DSlrels only capture the number of words to keep in mind until a suitable dependent or head is read or heard.

Nonetheless, this interpretation does not explain the whole picture with regards to dependency structure processing. As pointed out above, there is a significant relation between DSlrels and the left ATP, an area that is not traditionally considered part of the working memory network. Therefore, rather than interpreting these results as suggesting that dependency measures simply capture working memory loads imposed by the number of words to integrate into the parse, it might be more accurate to claim that, while dependency-related activity in the left ATP indeed computes sentence-level structural analyses, activity in areas such as the cingulate and the frontal and inferior parietal cortex might be well explained in terms of processing memory support to the activity in the anterior temporal and the inferior frontal regions. Further work is required to shed light on this possibility.

### Interaction Between Areas

The PPI analyses were conducted in order to see what type of interaction exists between these syntax processing areas (left ATP, IFG, and STG) and the rest of the brain.

The fact that activity in the left STG, modulated by PSxps, seems to drive the activation in a small portion of the left IFG, besides a large network of bilateral central and precentral regions, might indicate that there is an interaction between phrase-structure and dependency-structure processing areas. This might be supported also by the observation that IFG activity, modulated by DSlrels, explains activity in the left posterior perisylvian cortex (i.e., AG), and in a small portion of the right middle STG. Nonetheless these results cannot allow us to strongly claim a causal interaction between these sets of areas.

### Difference Between ROI and Voxel-wise Results

Part of the discrepancies between the ROI analyses, on the one hand, and the voxel-wise analyses, on the other, are likely due to statistical thresholding in the latter, which is, in many ways, a statistically insensitive method (as compared to ROI analyses). In the ROI analyses, we assess whether adding a syntactic predictor improves the linear model fit on the average activity in an anatomically defined brain area. The whole-brain analyses test whether the beta-coefficient of a regressor fits the activity of a single voxel in a statistically significant manner. Given that this is done on many voxels at once, there is a substantial risk of false negatives. The PPI analyses investigate the coefficients of the syntactic regressor convolved with the BOLD activity of a seed area while controlling for the BOLD activity of the area itself. Both in spirit and implementation, this is complementary to the activation-based approach taken in the ROI and whole-brain analyses. Differences are, therefore, to be expected.

### Conclusions

In this article we investigated whether different brain regions are sensitive to different kinds of syntactic operations. In order to do so, we assessed dependency and phrase-structure descriptors of sentences as predictors of brain activity in the left ATP, the left IFG, and the left STG—areas engaged during language processing.

We found that activity in the left ATP is better explained by dependency measures as compared to phrase-structure measures. These results differ from previous studies adopting phrase structures as the formalism of choice to characterize natural language syntax (Nelson et al., 2017). Our results are related to the ones presented by Brennan et al. (2016). They predicted fMRI data in both the left anterior and posterior portions of the temporal cortex during narrative listening using syntactic metrics derived from the phrase structure of the stimuli. In a more recent study, Li and Hale (2019) observed that a measure combining dependency and phrasal information significantly explains activity in the right anterior and left posterior temporal cortex. Nonetheless, two aspects distinguish our study from the ones by Brennan et al. (2016) and Li and Hale (2019). First, in our study we deliberately decided to keep phrasal and dependency measures apart under the assumption that their structural differences might explain activity in different areas composing the language network in the brain. As a matter of fact, our results show how the left ATP and the left STG are selective for one or the other. Second, our results, obtained on Dutch instead of English, may be taken to support the cross-linguistic validity of these observations, keeping in mind, nonetheless, that Dutch and English are closely related.

Our observations regarding the role of the left ATP are also in line with studies such as Westerlund et al. (2015) that show how this area is sensitive to a wide range of compositional structures, including verb-argument and preposition-argument pairs. We think that our results support the idea that ATP works as a hub for sentential-level semantic composition where words are combined according to the argument structure of the sentences, as captured by its dependency parse.

We also conducted PPI analyses investigating how the interaction between each of our ROIs and the rest of the brain is modulated by its preferred structural description as from the previous lme-analysis. We observed that the activity in the left STG, modulated by the number of closed phrase structures, might drive the activity in the left ATP. These results, while confirming a division of labour between brain regions, seems to point to an ancillary role of the STG and phrase-structure building subserving the dependency-style analysis that an area such ATP seems to perform. Further research is needed in order to investigate this interaction. A crucial point would be to assess whether the left STG sensitivity to PSG manifests at earlier latencies as compared to the sensitivity of the ATP to DG.

## ACKNOWLEDGMENTS

The work presented here was funded by the Netherlands Organisation for Scientific Research (NWO) Gravitation Grant 024.001.006 to the Language in Interaction Consortium.

## FUNDING INFORMATION

Antal van den Bosch, Nederlandse Organisatie voor Wetenschappelijk Onderzoek (http://dx.doi.org/10.13039/501100003246), Award ID: 024.001.006.

## AUTHOR CONTRIBUTIONS

**Alessandro Lopopolo**: Conceptualization: lead; Data curation: equal; Formal analysis: lead; Investigation: lead; Methodology: lead; Software: lead; Visualization: lead; Writing – original draft: lead; Writing – review & editing: lead. **Antal van den Bosch**: Formal analysis: equal; Funding acquisition: equal; Supervision: equal; Writing – review & editing: equal. **Karl-Magnus Petersson**: Supervision: equal; Writing – review & editing: equal. **Roel M. Willems**: Conceptualization: equal; Investigation: supporting; Methodology: supporting; Resources: equal; Supervision: equal; Writing – review & editing: equal.

## TECHNICAL TERMS

Phrase-structure grammar (PSG): Formalism describing the structure of a sentence by grouping words (terminal nodes) into phrases (non-terminal nodes), and phrases into higher-level phrases.
Dependency grammar (DG): Formalism describing the structure of a sentence by assigning binary head-dependent relations between pairs of words.
Terminal nodes: The surface form elements in the sentence structure according to PSG (i.e., the words of the sentence).
Parser: A software that computes the structure (the parse) of a sentence according to a grammar.

## References

[bib1] Baron, S. G. , & Osherson, D. N. (2011). Evidence for conceptual combination in the left anterior temporal lobe. NeuroImage, 55, 1847–1852. DOI: https://doi.org/10.1016/j.neuroimage.2011.01.066, PMID: 21281723 21281723 10.1016/j.neuroimage.2011.01.066

[bib2] Baron, S. G. , Thompson-Schill, S. L. , Weber, M. , & Osherson, D. (2010). An early stage of conceptual combination: Superimposition of constituent concepts in left anterolateral temporal lobe. Cognitive Neuroscience, 1(1), 44–51. DOI: https://doi.org/10.1080/17588920903548751, PMID: 24168244 24168244 10.1080/17588920903548751

[bib3] Bastiaanse, R. , Bouma, G. , & Post, W. (2009). Linguistic complexity and frequency in agrammatic speech production. Brain and Language, 109(1), 18–28. DOI: https://doi.org/10.1016/j.bandl.2008.12.004, PMID: 19217151 19217151 10.1016/j.bandl.2008.12.004

[bib4] Bemis, D. K. , & Pylkkänen, L. (2011). Simple composition: A magnetoencephalography investigation into the comprehension of minimal linguistic phrases. Journal of Neuroscience, 31(8), 2801–2814. DOI: https://doi.org/10.1523/JNEUROSCI.5003-10.2011, PMID: 21414902, PMCID: PMC6623787 21414902 10.1523/JNEUROSCI.5003-10.2011 PMC6623787

[bib5] Bemis, D. K. , & Pylkkänen, L. (2013). Basic linguistic composition recruits the left anterior temporal lobe and left angular gyrus during both listening and reading. Cerebral Cortex, 23(8), 1859–1873. DOI: https://doi.org/10.1093/cercor/bhs170, PMID: 22735156 22735156 10.1093/cercor/bhs170

[bib6] Bonhage, C. E. , Fiebach, C. J. , Bahlmann, J. , & Mueller, J. L. (2014). Brain signature of working memory for sentence structure: Enriched encoding and facilitated maintenance. Journal of Cognitive Neuroscience, 26, 1654–1671. DOI: https://doi.org/10.1162/jocn_a_00566, PMID: 24405186 24405186 10.1162/jocn_a_00566

[bib7] Borsley, R. D. (1998). Syntactic theory: A unified approach (2nd ed.). (Previous ed.: 1991.) E. Arnold.

[bib8] Brennan, J. R. , Stabler, E. P. , Van Wagenen, S. E. , Luh, W.-M. , & Hale, J. T. (2016). Abstract linguistic structure correlates with temporal activity during naturalistic comprehension. Brain and Language, 157–158, 81–94. DOI: https://doi.org/10.1016/j.bandl.2016.04.008, PMID: 27208858, PMCID: PMC4893969 10.1016/j.bandl.2016.04.008 PMC4893969 27208858

[bib9] Bright, P. , Moss, H. E. , Stamatakis, E. A. , & Tyler, L. K. (2005). The anatomy of object processing: The role of anteromedial temporal cortex. The Quarterly Journal of Experimental Psychology Section B, 58(3–4b), 361–377. DOI: https://doi.org/10.1080/02724990544000013, PMID: 16194974 10.1080/02724990544000013 16194974

[bib10] Brouwer, H. , Fitz, H. , & Hoeks, J. C. J. (2010). Modeling the noun phrase versus sentence coordination ambiguity in Dutch: Evidence from surprisal theory. In J. T. Hale (Ed.), Proceedings of the 2010 workshop on cognitive modeling and computational linguistics (pp. 72–80). Association for Computational Linguistics.

[bib11] Caramazza, A. , & Zurif, E. B. (1976). Dissociation of algorithmic and heuristic processes in language comprehension: Evidence from aphasia. Brain and Language, 3(4), 572–582. DOI: 10.1016/0093-934X(76)90048-1 974731

[bib12] Chomsky, N. (1957). Syntactic structures. Mouton and Co. DOI: 10.1515/9783112316009

[bib13] Chomsky, N. (1965). Aspects of the theory of syntax. The MIT Press. http://www.amazon.com/Aspects-Theory-Syntax-Noam-Chomsky/dp/0262530074. DOI: 10.21236/AD0616323

[bib14] D’Esposito, M. , & Postle, B. R. (2007). The cognitive neuroscience of working memory. Annual Review of Psychology, 66, 115–142. DOI: https://doi.org/10.1146/annurev-psych-010814-015031, PMID: 25251486, PMCID: PMC4374359 10.1146/annurev-psych-010814-015031 PMC4374359 25251486

[bib15] Dronkers, N. F. , Wilkins, D. P. , Van Valin, R. D. J. , Redfern, B. B. , & Jaeger, J. J. (2004). Lesion analysis of the brain areas involved in language comprehension. Cognition, 92(1–2), 145–177. DOI: https://doi.org/10.1016/j.cognition.2003.11.002, PMID: 15037129 15037129 10.1016/j.cognition.2003.11.002

[bib16] Frank, S. L. , Bod, R. , & Christiansen, M. H. (2012). How hierarchical is language use? Proceedings of the Royal Society B: Biological Sciences, 279. DOI: https://doi.org/10.1098/rspb.2012.1741, PMID: 22977157, PMCID: PMC3479729 10.1098/rspb.2012.1741 PMC3479729 22977157

[bib17] Friederici, A. D. , Fiebach, C. J. , Schlesewsky, M. , Bornkessel, I. , & von Cramon, D. Y. (2005). Processing linguistic complexity and grammaticality in the left frontal cortex. Cerebral Cortex, 16(12), 1709–1717. DOI: https://doi.org/10.1093/cercor/bhj106, PMID: 16400163 10.1093/cercor/bhj106 16400163

[bib18] Friederici, A. D. , Meyer, M. , & von Cramon, D. Y. (2000). Auditory language comprehension: An event-related fMRI study on the processing of syntactic and lexical information. Brain and Language, 74, 289–300. DOI: https://doi.org/10.1006/brln.2000.2313, PMID: 10950920 10950920 10.1006/brln.2000.2313

[bib20] Friston, K. (2011). Functional and effective connectivity: A review. Brain Connectivity, 1, 13–36. DOI: https://doi.org/10.1089/brain.2011.0008, PMID: 22432952 22432952 10.1089/brain.2011.0008

[bib21] Friston, K. J. , Büchel, C. , Fink, G. R. , Morris, J. , & Dolan, R. J. (1997). Psychophysiological and modulatory interactions in neuroimaging. NeuroImage, 6, 218–229. DOI: https://doi.org/10.1006/nimg.1997.0291, PMID: 9344826 9344826 10.1006/nimg.1997.0291

[bib19] Friston, K. J. , Holmes, A. P. , Poline, J.-B. , Grasby, P. J. , Williams, S. C. R. , Frackowiak, R. S. J. , & Turner, R. (1995). Analysis of fMRI time-series revisited. NeuroImage, 2(1), 45–53. DOI: https://doi.org/10.1006/nimg.1995.1007, PMID: 9343589 9343589 10.1006/nimg.1995.1007

[bib22] Gauthier, I. , Anderson, A. W. , Tarr, M. J. , Skudlarski, P. , & Gore, J. C. (1997). Levels of categorization in visual recognition studied using functional magnetic resonance imaging. Current Biology, 7(9), 645–651. DOI: 10.1016/S0960-9822(06)00291-0 9285718

[bib23] Hagoort, P. (2005). On Broca, brain, and binding: A new framework. Trends in Cognitive Sciences, 9(9), 416–423. DOI: https://doi.org/10.1016/j.tics.2005.07.004, PMID: 16054419 16054419 10.1016/j.tics.2005.07.004

[bib24] Hagoort, P. (2013). MUC (Memory, Unification, Control) and beyond. Frontiers in Psychology, 4, 416. DOI: https://doi.org/10.3389/fpsyg.2013.00416, PMID: 23874313, PMCID: PMC3709422 23874313 10.3389/fpsyg.2013.00416 PMC3709422

[bib25] Hays, D. G. (1964). Dependency theory: A formalism and some observations (Memorandum RM-4087-PR). United States Air Force Project Rand. https://www.rand.org/content/dam/rand/pubs/research_memoranda/2008/RM4087.pdf. DOI: 10.2307/411934 24547306

[bib26] Hodges, J. R. , Graham, N. , & Patterson, K. (1995). Charting the progression in semantic dementia: Implications for the organisation of semantic memory. Memory, 3(3–4), 463–495. DOI: https://doi.org/10.1080/09658219508253161, PMID: 8574874 8574874 10.1080/09658219508253161

[bib27] Hodges, J. R. , Patterson, K. , Oxbury, S. , & Funnell, E. (1992). Semantic dementia: Progressive fluent aphasia with temporal lobe atrophy. Brain, 115(6), 1783–1806. DOI: https://doi.org/10.1093/brain/115.6.1783, PMID: 1486461 1486461 10.1093/brain/115.6.1783

[bib28] Humphries, C. , Binder, J. R. , Medler, D. A. , & Liebenthal, E. (2006). Syntactic and semantic modulation of neural activity during auditory sentence comprehension. Journal of Cognitive Neuroscience, 18(4), 665–679. DOI: https://doi.org/10.1162/jocn.2006.18.4.665, PMID: 16768368, PMCID: PMC1635792 16768368 10.1162/jocn.2006.18.4.665 PMC1635792

[bib29] Humphries, C. , Binder, J. R. , Medler, D. A. , & Liebenthal, E. (2007). Time course of semantic processes during sentence comprehension: An fMRI study. NeuroImage, 36, 924–932. DOI: https://doi.org/10.1016/j.neuroimage.2007.03.059, PMID: 17500009, PMCID: PMC1941617 17500009 10.1016/j.neuroimage.2007.03.059 PMC1941617

[bib30] Jung, W.-Y. (1998). Syntaktische Relationen im Rahmen der Dependenzgrammatik. Buske.

[bib31] Keuleers, E. , Brysbaert, M. , & New, B. (2010). SUBTLEX-NL: A new measure for Dutch word frequency based on film subtitles. Behavior Research Methods, 42(3), 643–650. DOI: https://doi.org/10.3758/BRM.42.3.643, PMID: 20805586 20805586 10.3758/BRM.42.3.643

[bib32] Kos, M. , van den Brink, D. , & Hagoort, P. (2012). Individual variation in the late positive complex to semantic anomalies. Frontiers in Psychology, 3, 318. DOI: https://doi.org/10.3389/fpsyg.2012.00318, PMID: 22973249, PMCID: PMC3434872 22973249 10.3389/fpsyg.2012.00318 PMC3434872

[bib33] Kos, M. , van den Brink, D. , Snijders, T. M. , Rijpkema, M. , Franke, B. , Fernandez, G. , & Hagoort, P. (2012). CNTNAP2 and language processing in healthy individuals as measured with ERPs. PLOS ONE, 7, 1–8. DOI: https://doi.org/10.1371/journal.pone.0046995, PMID: 23115634, PMCID: PMC3480372 10.1371/journal.pone.0046995 PMC3480372 23115634

[bib34] Kos, M. , Vosse, T. , van Den Brink, D. , & Hagoort, P. (2010). About edible restaurants: Conflicts between syntax and semantics as revealed by ERPs. Frontiers in Psychology, 1, 222. DOI: https://doi.org/10.3389/fpsyg.2010.00222, PMID: 21833277, PMCID: PMC3153827 10.3389/fpsyg.2010.00222 PMC3153827 21833277

[bib35] Kübler, S. , McDonald, R. , & Nivre, J. (2009). Dependency parsing: Synthesis lectures on human language technologies. Morgan & Claypool Publishers. DOI: 10.2200/S00169ED1V01Y200901HLT002

[bib36] Li, J. , & Hale, J. (2019). Grammatical predictors for fMRI time-courses. In R. C. Berwick & E. P. Stabler (Eds.), Minimalist parsing (pp. 159–173). Oxford Scholarship. DOI: 10.1093/oso/9780198795087.003.0007

[bib37] Linebarger, M. C. , Schwartz, M. F. , & Saffran, E. M. (1983). Sensitivity to grammatical structure in so-called agrammatic aphasics. Cognition, 13(3), 361–392. DOI: 10.1016/0010-0277(83)90015-X 6683142

[bib38] Lopopolo, A. , Frank, S. L. , van den Bosch, A. , Nijhof, A. , & Willems, R. M. (2018). The narrative brain dataset (NBD), an fMRI dataset for the study of natural language processing in the brain. In B. Devereux , E. Shutova , & C.-R. Huang (Eds.), Proceedings of LREC 2018 Workshop “Linguistic and Neuro-Cognitive Resources (LiNCR)” (pp. 8–11). European Language Resources Association.

[bib39] Lopopolo, A. , Frank, S. L. , van den Bosch, A. , & Willems, R. M. (2017). Using stochastic language models (SLM) to map lexical, syntactic, and phonological information processing in the brain. PLOS ONE, 12(5), e0177794. DOI: https://doi.org/10.1371/journal.pone.0177794, PMID: 28542396, PMCID: PMC5436813 28542396 10.1371/journal.pone.0177794 PMC5436813

[bib40] Lopopolo, A. , Frank, S. L. , van den Bosch, A. , & Willems, R. (2019). Dependency parsing with your eyes: Dependency structure predicts eye regressions during reading. In Proceedings of the workshop on cognitive modeling and computational linguistics (pp. 77–85). Association for Computational Linguistics. DOI: 10.18653/v1/W19-2909

[bib41] Matchin, W. , Hammerly, C. , & Lau, E. (2017). The role of the IFG and pSTS in syntactic prediction: Evidence from a parametric study of hierarchical structure in fMRI. Cortex, 88, 106–123. DOI: https://doi.org/10.1016/j.cortex.2016.12.010, PMID: 28088041 28088041 10.1016/j.cortex.2016.12.010

[bib42] Mazoyer, B. M. , Tzourio, N. , Frak, V. , Syrota, A. , Murayama, N. , Levrier, O. , Salamon, G. , Dehaene, S. , Cohen, L. , & Mehler, J. (1993). The cortical representation of speech. Journal of Cognitive Neuroscience, 5(4), 467–479. DOI: https://doi.org/10.1162/jocn.1993.5.4.467, PMID: 23964919 23964919 10.1162/jocn.1993.5.4.467

[bib43] Mel’cˇuk, I. (1988). Dependency syntax: Theory and practice. State University of New York Press.

[bib44] Moss, H. , Rodd, J. , Stamatakis, E. , Bright, P. , & Tyler, L. K. (2004). Anteromedial temporal cortex supports fine-grained differentiation among objects. Cerebral Cortex, 15(5), 616–627. DOI: https://doi.org/10.1093/cercor/bhh163, PMID: 15342435 15342435 10.1093/cercor/bhh163

[bib45] Mummery, C. J. , Patterson, K. , Veltman, D. J. , Ashburner, J. , Frackowiak, R. S. , & Hodges, J. R. (2000). A voxel-based morphometry study of semantic dementia: Relationship between temporal lobe atrophy and semantic memory. Annals of Neurology, 47(1), 36–45. DOI: 10.1002/1531-8249(200001)47:1<36::AID-ANA8>3.0.CO;2-L 10632099

[bib46] Nee, D. E. , & D’Esposito, M. (2016). The hierarchical organization of the lateral prefrontal cortex. eLife, 5. DOI: https://doi.org/10.7554/eLife.12112, PMID: 26999822, PMCID: PMC4811776 10.7554/eLife.12112 PMC4811776 26999822

[bib47] Nelson, M. J. , El Karoui, I. , Giber, K. , Yang, X. , Cohen, L. , Koopman, H. , Cash, S. S. , Naccache, L. , Hale, J. T. , Pallier, C. , & Dehaene, S. (2017). Neurophysiological dynamics of phrase-structure building during sentence processing. Proceedings of the National Academy of Sciences, 114(18). DOI: https://doi.org/10.1073/pnas.1701590114, PMID: 28416691, PMCID: PMC5422821 10.1073/pnas.1701590114 PMC5422821 28416691

[bib48] Oostdijk, N. (2000). The spoken Dutch corpus. Overview and first evaluation. In Proceedings of the Second International Conference on Language Resources and Evaluation (LREC’00). European Language Resources Association (ELRA). Retrieved from http://www.lrec-conf.org/proceedings/lrec2000/pdf/110.pdf

[bib49] O’Reilly, J. X. , Woolrich, M. W. , Behrens, T. E. , Smith, S. M. , & Johansen-Berg, H. (2012). Tools of the trade: Psychophysiological interactions and functional connectivity. Social Cognitive and Affective Neuroscience, 7(5), 604–609. DOI: https://doi.org/10.1093/scan/nss055, PMID: 22569188, PMCID: PMC3375893 22569188 10.1093/scan/nss055 PMC3375893

[bib50] Pallier, C. , Devauchelle, A.-D. , & Dehaene, S. (2011). Cortical representation of the constituent structure of sentences. Proceedings of the National Academy of Sciences, 108(6), 2522–2527. DOI: https://doi.org/10.1073/pnas.1018711108, PMID: 21224415, PMCID: PMC3038732 10.1073/pnas.1018711108 PMC3038732 21224415

[bib51] Patterson, K. , Nestor, P. J. , & Rogers, T. T. (2007). Where do you know what you know? The representation of semantic knowledge in the human brain. Nature Reviews Neuroscience, 8, 976–987. DOI: https://doi.org/10.1038/nrn2277, PMID: 18026167 18026167 10.1038/nrn2277

[bib52] Poser, B. , Koopmans, P. , Witzel, T. , Wald, L. , & Barth, M. (2010). Three dimensional echo-planar imaging at 7 Tesla. NeuroImage, 51(1), 261–266. DOI: https://doi.org/10.1016/j.neuroimage.2010.01.108, PMID: 20139009, PMCID: PMC2853246 20139009 10.1016/j.neuroimage.2010.01.108 PMC2853246

[bib53] Rambow, O. (2010). The simple truth about dependency and phrase structure representations: An opinion piece. In Human language technologies: The 2010 annual conference of the North American Chapter of the Association for Computational Linguistics (pp. 337–340). Association for Computational Linguistics.

[bib54] Rogalsky, C. , & Hickok, G. (2008). Selective attention to semantic and syntactic features modulates sentence processing networks in anterior temporal cortex. Cerebral Cortex, 19(4), 786–796. DOI: https://doi.org/10.1093/cercor/bhn126, PMID: 18669589, PMCID: PMC2651476 18669589 10.1093/cercor/bhn126 PMC2651476

[bib55] Rogers, T. T. , Hocking, J. , Noppeney, U. , Mechelli, A. , Gorno-Tempini, M. L. , Patterson, K. , & Price, C. J. (2006). Anterior temporal cortex and semantic memory: Reconciling findings from neuropsychology and functional imaging. Cognitive, Affective, & Behavioral Neuroscience, 6(3), 201–213. DOI: https://doi.org/10.3758/CABN.6.3.201, PMID: 17243356 10.3758/cabn.6.3.201 17243356

[bib56] Rogers, T. T. , Ralph, M. A. L. , Garrard, P. , Bozeat, S. , McClelland, J. L. , Hodges, J. R. , & Patterson, K. (2004). Structure and deterioration of semantic memory: A neuropsychological and computational investigation. Psychological Review, 111(1), 205–235. DOI: https://doi.org/10.1037/0033-295X.111.1.205, PMID: 14756594 14756594 10.1037/0033-295X.111.1.205

[bib57] Schäfer, R. , & Bildhauer, F. (2012). Building large corpora from the web using a new efficient tool chain. In N. Calzolari , K. Choukri , T. Declerck , M. U. Dogan , B. Maegaard , J. Mariani , J. Odijk , & S. Piperidis (Eds.), Proceedings of the eighth international conference on language resources and evaluation, LREC 2012 (pp. 486–493). European Language Resources Association (ELRA). Retrieved from https://dblp.uni-trier.de/db/conf/lrec/lrec2012.html#SchaferB12

[bib58] Schneider, G. (1998). A linguistic comparison of constituency, dependency and link grammar. [Master’s thesis] University of Zurich.

[bib59] Slotnick, S. D. , Moo, L. R. , Segal, J. B. , & Hart, J. J. (2003). Distinct prefrontal cortex activity associated with item memory and source memory for visual shapes. Cognitive Brain Research, 17(1), 75–82. DOI: 10.1016/S0926-6410(03)00082-X 12763194

[bib60] Snijders, T. M. , Vosse, T. , Kempen, G. , Van Berkum, J. J. , Petersson, K. M. , & Hagoort, P. (2008). Retrieval and unification of syntactic structure in sentence comprehension: An fMRI study using word-category ambiguity. Cerebral Cortex, 19(7), 1493–1503. DOI: https://doi.org/10.1093/cercor/bhn187, PMID: 19001084 19001084 10.1093/cercor/bhn187

[bib61] Stolcke, A. (2002). SRILM—An extensible language modeling toolkit. In Proceedings of the 7th international conference on spoken language processing (ICSLP 2002) (pp. 901–904).

[bib62] Stowe, L. A. , Broere, C. A. J. , Paans, A. M. J. , Wijers, A. A. , Mulder, G. , Vaalburg, W. , & Zwarts, F. (1998). Localizing components of a complex task: Sentence processing and working memory. NeuroReport, 9(13), 2995–2999. DOI: https://doi.org/10.1097/00001756-199809140-00014, PMID: 9804304 9804304 10.1097/00001756-199809140-00014

[bib63] Tesnière, L. (2015). Elements of structural syntax ( T. Osborne & S. Kahane , Trans). John Benjamins Publishing Company. Retrieved from https://books.google.nl/books?id=FNjooAEACAAJ . DOI: 10.1075/z.185

[bib64] Thothathiri, M. , Kimberg, D. Y. , & Schwartz, M. F. (2012). The neural basis of reversible sentence comprehension: Evidence from voxel-based lesion symptom mapping in aphasia. Journal of Cognitive Neuroscience, 24(1), 212–222. DOI: https://doi.org/10.1162/jocn_a_00118, PMID: 21861679, PMCID: PMC3389786 21861679 10.1162/jocn_a_00118 PMC3389786

[bib65] Tyler, L. K. , Randall, B. , & Stamatakis, E. A. (2008). Cortical differentiation for nouns and verbs depends on grammatical markers. Journal of Cognitive Neuroscience, 20(8), 1381–1389. DOI: https://doi.org/10.1162/jocn.2008.20095, PMID: 18303983 18303983 10.1162/jocn.2008.20095

[bib66] Tyler, L. K. , Stamatakis, E. A. , Bright, P. , Acres, K. , Abdallah, S. , Rodd, J. M. , & Moss, H. E. (2004). Processing objects at different levels of specificity. Journal of Cognitive Neuroscience, 16, 351–362. DOI: https://doi.org/10.1162/089892904322926692, PMID: 15072671 15072671 10.1162/089892904322926692

[bib67] Tzourio-Mazoyer, N. , Landeau, B. , Papathanassiou, D. , Crivello, F. , & Joliot, M. (2001). Automated anatomical labeling of activations in SPM using a macroscopic anatomical parcellation of the MNI MRI single-subject brain. NeuroImage, 15, 273–289. DOI: https://doi.org/10.1006/nimg.2001.0978, PMID: 11771995 10.1006/nimg.2001.0978 11771995

[bib68] van der Beek, L. , Bouma, G. , Malouf, R. , & van Noord, G. (2002). The Alpino Dependency Treebank. In Computational linguistics in the Netherlands 2001. Brill. DOI: 10.1163/9789004334038_003

[bib69] van Noord, G. J. M. (2006). At last parsing is now operational. In P. Mertens , C. Fairon , A. Dister , & P. Watrin (Eds.), TALN06. Verbum Ex Machina. Actes de la 13e conference sur le traitement automatique des langues naturelles (pp. 20–42). Leuven University Press.

[bib70] Westerlund, M. , Kastner, I. , Kaabi, M. A. , & Pylkkänen, L. (2015). The LATL as locus of composition: MEG evidence from English and Arabic. Brain and Language, 141, 124–134. DOI: https://doi.org/10.1016/j.bandl.2014.12.003, PMID: 25585277 25585277 10.1016/j.bandl.2014.12.003

[bib71] Westerlund, M. , & Pylkkänen, L. (2014). The role of the left anterior temporal lobe in semantic composition vs. semantic memory. Neuropsychologia, 57, 59–70. DOI: https://doi.org/10.1016/j.neuropsychologia.2014.03.001, PMID: 24631260 24631260 10.1016/j.neuropsychologia.2014.03.001

[bib72] Willems, R. M. , Frank, S. L. , Nijhof, A. D. , Hagoort, P. , & Bosch, A. V. D. (2016). Prediction during natural language comprehension. Cerebral Cortex, 26(6), 2506–2516. DOI: https://doi.org/10.1093/cercor/bhv075, PMID: 25903464 25903464 10.1093/cercor/bhv075

[bib73] Wulfeck, B. , & Bates, E. (1991). Differential sensitivity to errors of agreement and word order in Broca’s aphasia. Journal of Cognitive Neuroscience, 3(3), 258–272. DOI: https://doi.org/10.1162/jocn.1991.3.3.258, PMID: 23964841 23964841 10.1162/jocn.1991.3.3.258

[bib74] Zaccarella, E. , & Friederici, A. D. (2015). Merge in the human brain: A sub-region based functional investigation in the left pars opercularis. Frontiers in Psychology, 6, 1818. DOI: https://doi.org/10.3389/fpsyg.2015.01818, PMID: 26640453, PMCID: PMC4661288 26640453 10.3389/fpsyg.2015.01818 PMC4661288

[bib75] Zaccarella, E. , Meyer, L. , Makuuchi, M. , & Friederici, A. D. (2015). Building by syntax: The neural basis of minimal linguistic structures. Cerebral Cortex, 27(1), 411–421. DOI: https://doi.org/10.1093/cercor/bhv234, PMID: 26464476 10.1093/cercor/bhv234 26464476

